# Supplementary material for: Molecular basis for the enzymatic inactivity of class III glutaredoxin ROXY9 on standard glutathionylated substrates
Source: Nat Commun. 2025 Jan 11;16:589. doi: 10.1038/s41467-024-55532-z (PMC11724882; doi:10.1038/s41467-024-55532-z)
Supplement: Supplementary file 1 — Supplementary Information [file 41467_2024_55532_MOESM1_ESM.pdf]

Supplementary Figures 1-19
and Supplementary Tables 1-2

Supplementary Figure 1

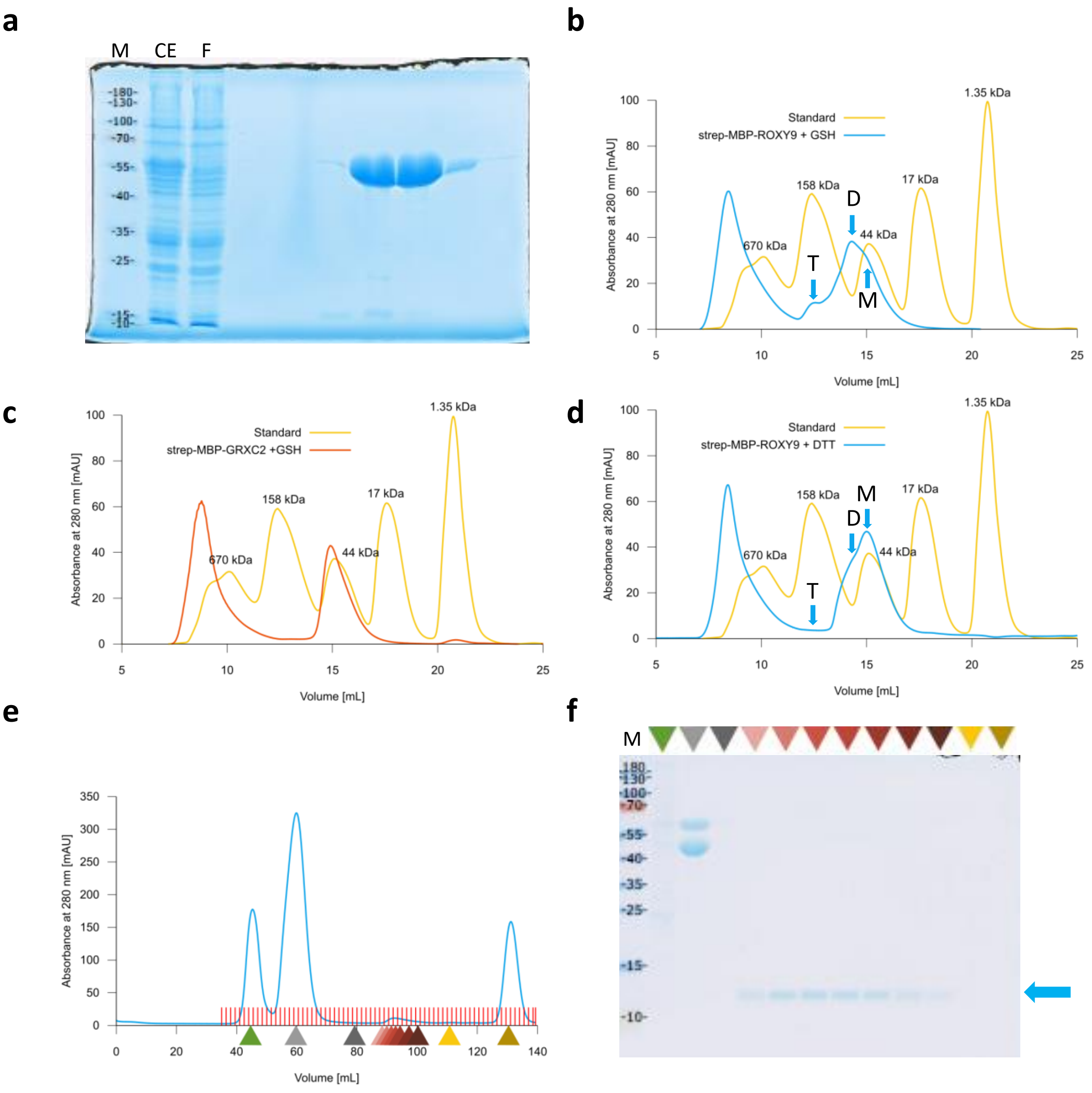

Supplementary Figure 1. Purification, TEV cleavage and gel filtration of ROXY9 a Cell extracts (CE) from *T. ni* Hi5 insect cells expressing strep-MBP-ROXY9 were loaded onto a MBPTrap column connected to an Äkta prime chromatography system. The flowthrough f and selected fractions were analysed by PAGE (12%). **b-d** Overlays of the gel filtration chromatograms (Superdex 20010/300 GL column) of strep-MBP-ROXY9 (blue) and strep-MBP-GRXC2 (orange) and calibration runs (yellow). Numbers above the peaks indicate the molecular weights of the standard proteins. The theoretical values of the strep-MBP-ROXY9 and strep-MBP-GRXC2 monomers are 61 and 64.6 kDa, respectively. Blue arrows in **(b)** and **(d)** point at possible monomers (M), dimers (D) and trimers (T). Proteins were purified in the presence of 5 mM GSH, in **(d)** GSH was replaced by 1 mM DTT. **e** Gel filtration (Superdex S75) of strep-MBP-ROXY9 after TEV cleavage. The triangles denote protein fractions analysed by SDS PAGE (15%) shown in **(f).** The arrow points at the 12 kDa ROXY9 protein. The fraction marked with a grey triangle contains the cleaved and uncleaved MBP fusion protein. Numbers in lanes M denote the molecular mass of marker proteins in kDa.

Supplementary Figure 2

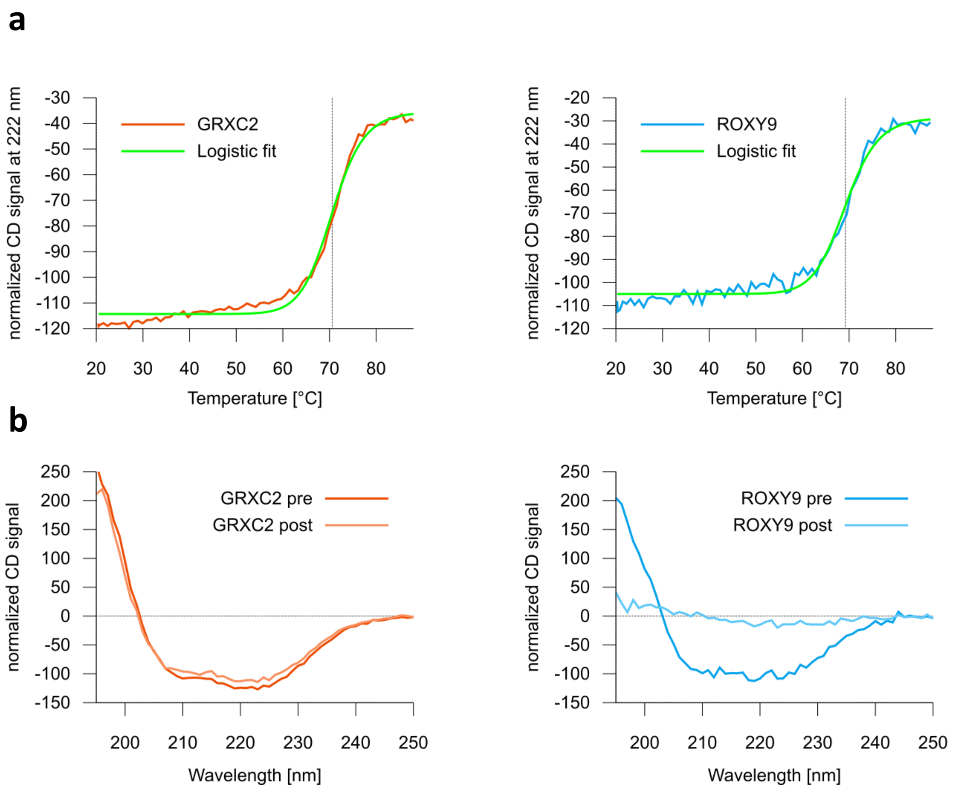

Supplementary Figure 2. Melting curves of ROXY9 and GRXC2 a GRXC2 and ROXY9 were heated and ellipticity was tracked at 222 nm. The respective Tms of 69.2ºC and 70.6ºC according to the logistic fits are indicated by the dashed lines. **b** CD spectra of ROXY9 and GRXC2 before and after heat treatment. Data sets before heat treatment are the same as in Fig. 1b (samples in 1 mM DTT).

Supplementary Figure 3

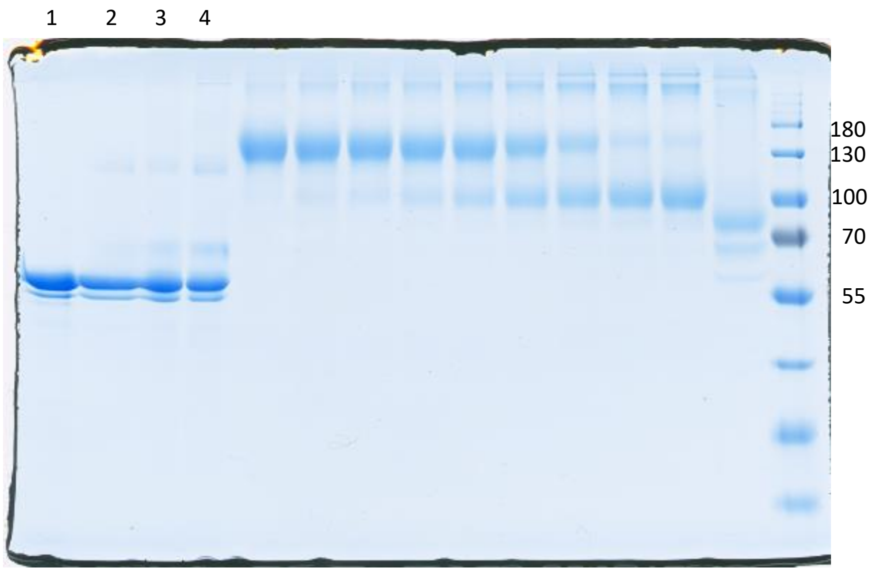

Supplementary Figure 3. Analysis of non-alkylated strep-MBP-ROXY9 on a non-reducing SDS PAGE

Lane 1: after purificiation in the presence of 5 mM GSH

Lane 2: after reduction with DTT and passage through a desalting column in the absence of any reduntant

Lane 3: after reduction with DTT, TCA precipitation and incubation in alkylation buffer without mmPEG

Lane 4. after oxidation with dithiane, TCA precipitation and incubation in alkylation buffer without mmPEG

The gel is the same as shown in Fig. 2b.

Supplementary Figure 4

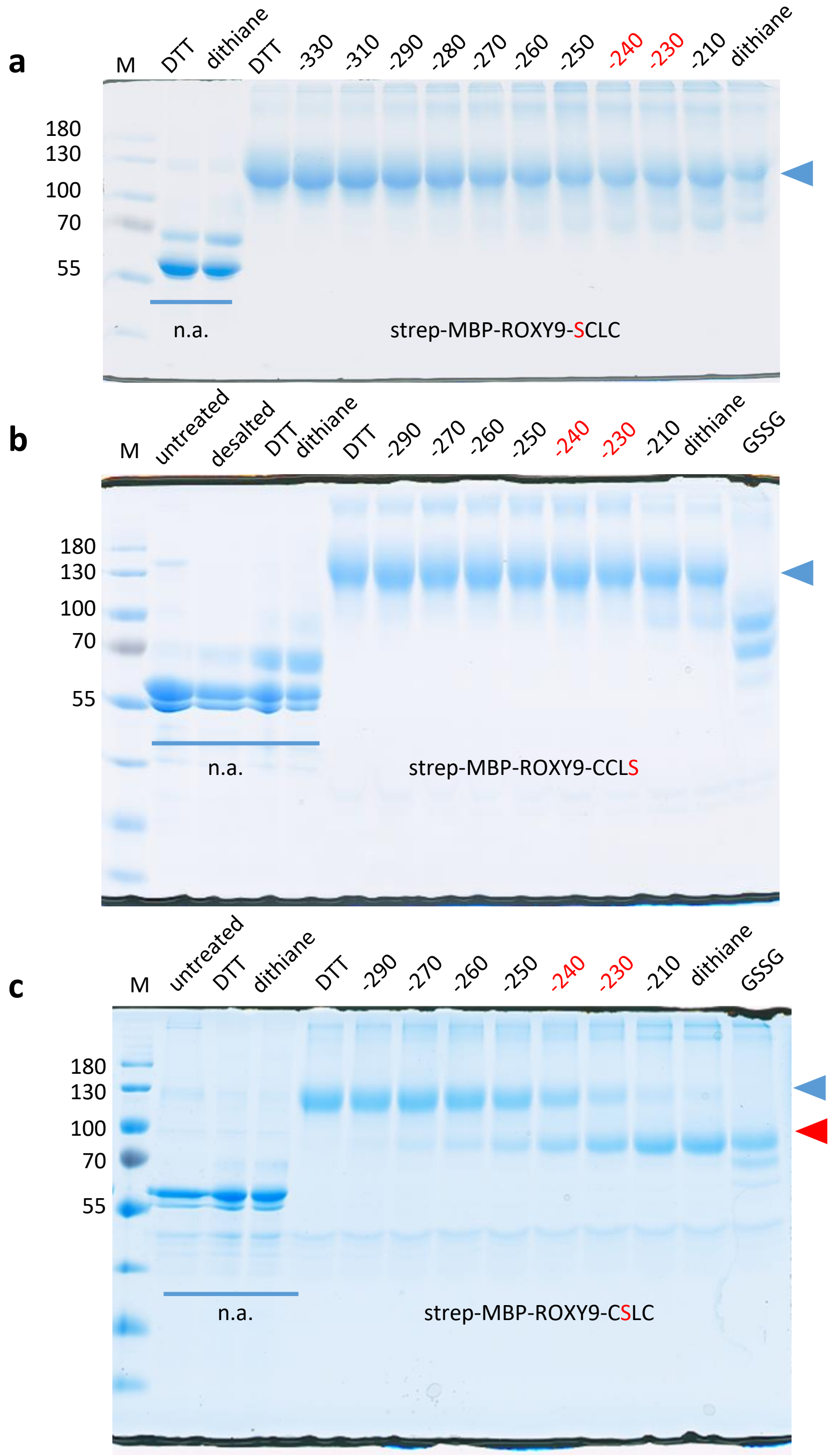

Supplementary Figure 4. Redox titration of strep-MBP-ROXY9 active site variants Strep-MBP-ROXY9-SCLC, strep-MBP-ROXY9-CCLS, and strep-MBP-ROXY9-CSLC were reduced with DTT, desalted and mixed with different DTT/dithiane redox buffers establishing the indicated redox potentials or with GSSG. After TCA precipitation, reduced cysteines were labelled with 5 kDa mmPEG. Samples were separated by non-reducing SDS PAGE. Midpoint redox potentials as observed for the wild-type protein (Fig. 2) are indicated in red. The first lanes (n.a.) contain the respective protein without alkylation. The “untreated” and “desalted” proteins were loaded without any further manipulation, while the DTT- and dithiane-treated samples were subjected to the same procedure as the other samples but without mmPEG in the alkylation buffer. The blue triangle denotes the reduced state, the red triangle the disulfide bridge-containing state. The molecular mass marker was loaded in lane M and sizes in kDa are indicated.

Supplementary Figure 5

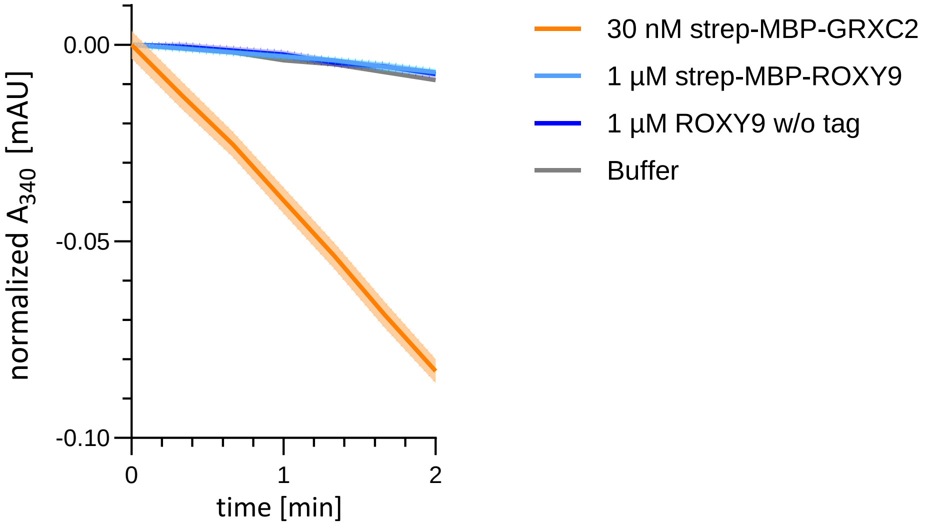

Supplementary Figure 5. HED assay of strep-MBP-ROXY9, strep-MBP-GRXC2 and ROXY9 Proteins were purified in the presence of 1 mM DTT and dialyzed against 1 mM GSH in 100 mM Na_2HPO_4/KH_2PO_4 p*H* 7.5. The final reaction mixtures (200 µl) contained 100 mM Na_2HPO_4/KH_2PO_4 p*H* 7.5, 0.2 mM NADPH, 1 mM GSH, 0.3 U/µl glutathione reductase and 1 mM HED. The consumption of NADPH was measured spectrophotometrically at 340 nm. Error bars represent the standard error of the mean resulting from three or two (ROXY9 w/o tag) technical replicates.

Supplementary Figure 6

a

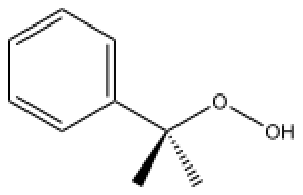

Cumene hydroperoxide

b

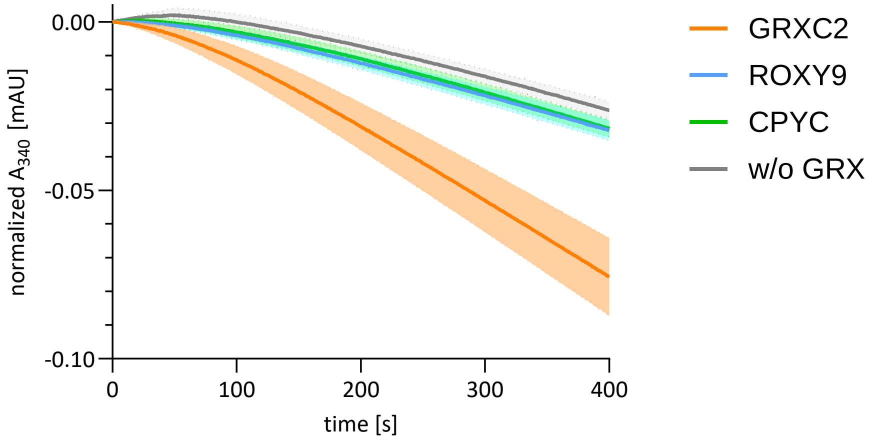

Supplementary Figure 6. CHP assay with strep-MBP-GRXC2, strep-MBP-ROXY9 and strep-MBP-ROXY9-CPYC a Structure of cumene hydroperoxide **b** GRX samples were pre-reduced with DTT and the buffer was exchanged for buffer (100 mM HEPES p*H* 7) containing 1 mM fresh GSH. GRX samples (100 µg) or GSH-containing buffer as control were added to a buffer containing 0.7 mM GSH, 0.28 mM NADPH and 0.3 U/µl glutathione reductase. After 60 s mixing and equilibration time, the reaction was started with the addition of the CHP substrate (final concentration: 1.5 mM). The change in absorbance at 340 nm was followed after an additional 30 s mixing and equilibration time for 16 min 40 s. Data are displayed as mean and standard deviation of three independent measurements belonging to one protein preparation.

Supplementary Figure 7

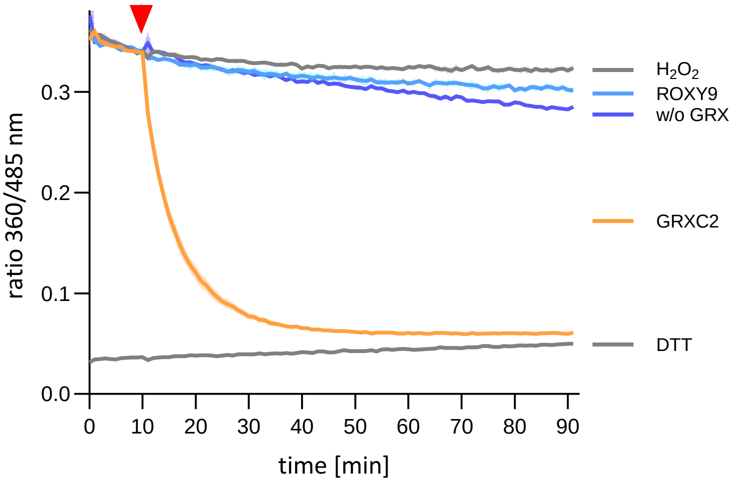

Supplementary Figure 7. Reductase activity of GRXC2 and ROXY9 with roGFP2 as a substrate Final concentrations were 2 µM roGFP2, 2 µM GRX and 2 mM GSH, which was added at 10 min (red triangle). References were treated with DTT or H_2O_2, respectively, to represent fully reduced or oxidized roGFP2. roGFP2 fluorescence was detected at 528 nm after excitation at 360 and 485 nm. Error bars represent the standard error of the mean resulting from three technical replicates.

Supplementary Figure 8

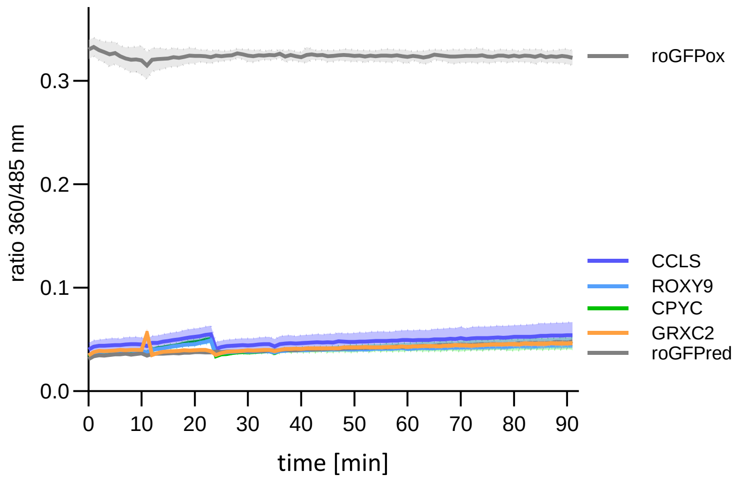

Supplementary Figure 8. roGFP2 assay with strep-MBP-GRXC2, strep-MBP-ROXY9 and strep-MBP-ROXY9-CPYC in the absence of GSSG Final concentrations were 2 µM roGFP2 and 2 µM GRX. roGFP2 incubated with 10 mM DTT or 10 mM H_2O_2 were included as references. roGFP2 fluorescence was detected at 528 nm after excitation at 360 and 485 nm. Error bars represent the standard error of the mean resulting from three technical replicates.

Supplementary Figure 9

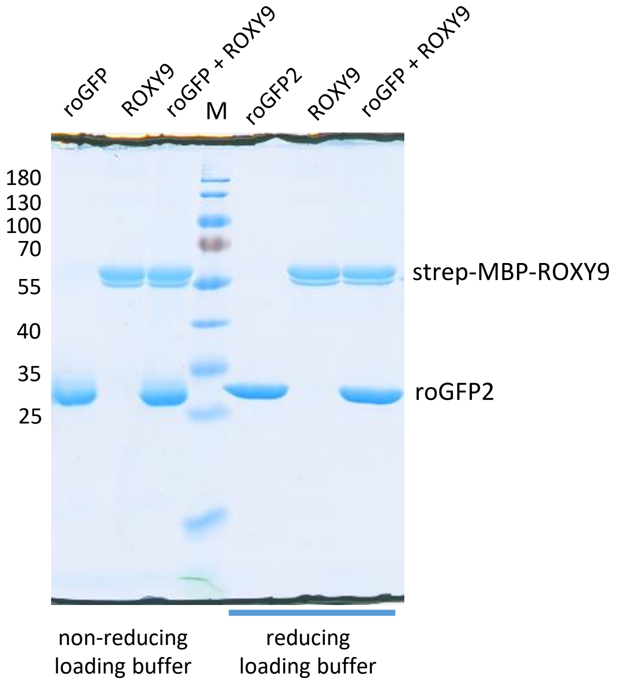

Supplementary Figure 9. SDS PAGE of strep-MBP-ROXY9 and roGFP2 co-incubated in the presence of GSSG Equimolar amounts of strep-MBP-ROXY9 and roGFP2 were incubated in the presence of 50 mM GSSG. After addition of either non-reducing (left part) or reducing loading buffer, samples were analysed by SDS PAGE (12%). The molecular mass marker was loaded in lane M and sizes in kDa are indicated on the left.

Supplementary Figure 10

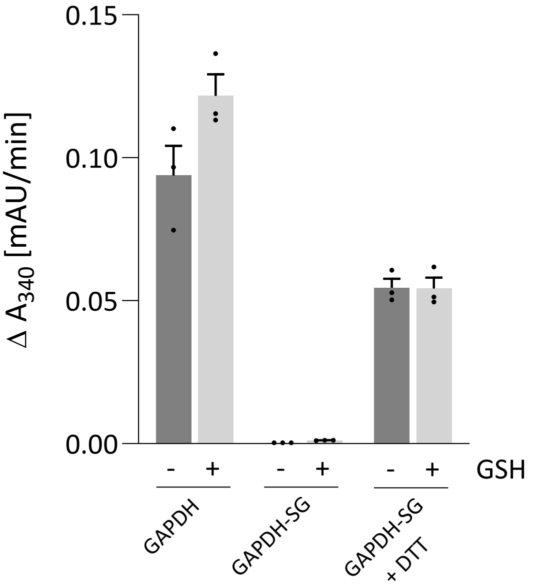

Supplementary Figure 10. Inactivation and reactivation of GAPDH activity Enzymatically inactive glutathionylated GAPDH (GAPDH-SG) (0.6 µM) was incubated for 30 min with DTT. Consumption of NADH per min upon reduction of 1,3 bisphosphoglycerate was monitored at 340 nm. Untreated GAPDH (0.6 µM) was used as control. Error bars represent the standard error of the mean resulting from three independent experiments. Four technical replicates were performed to calculate the mean of each independent measurement.

Supplementary Figure 11

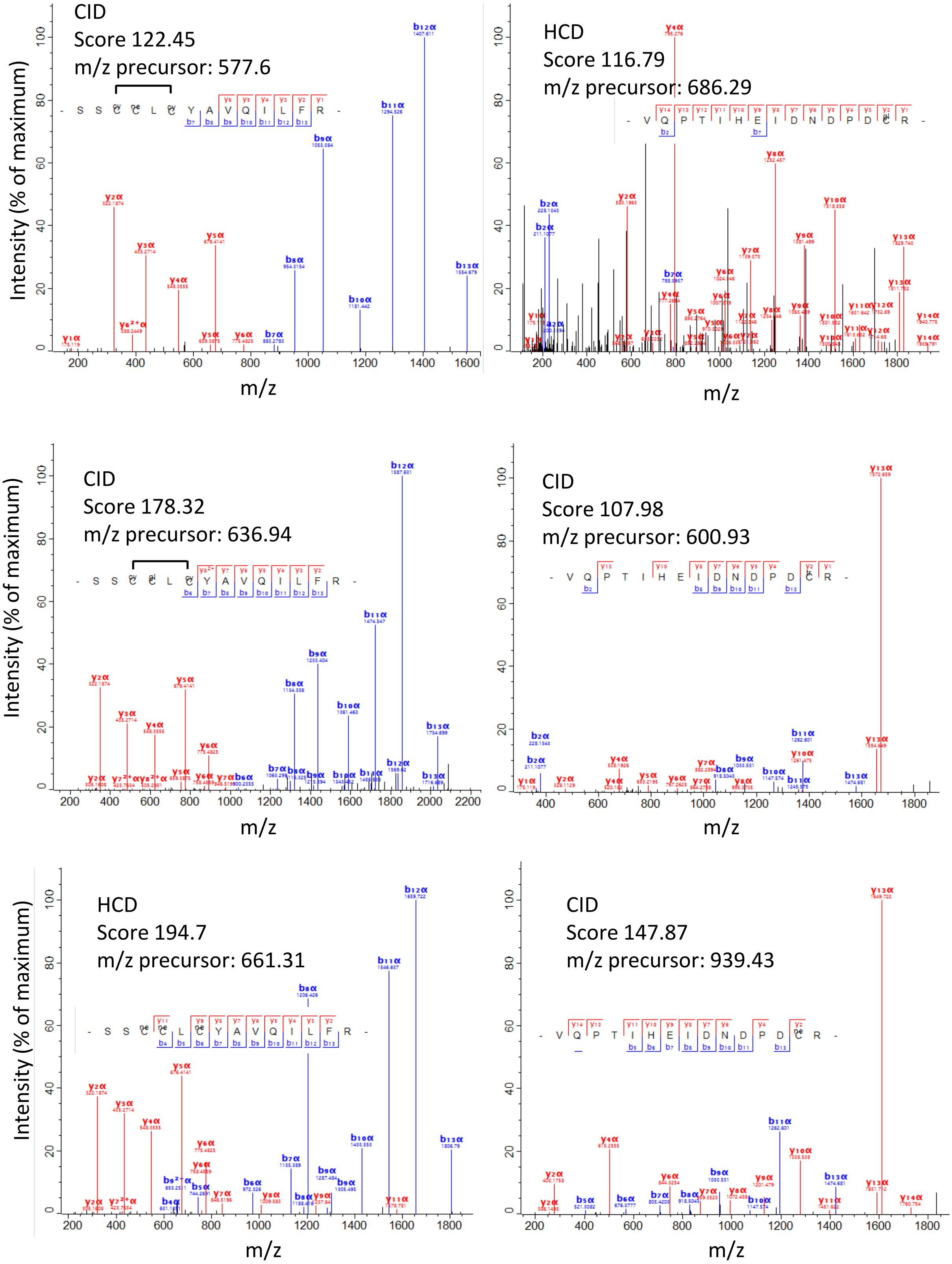

Supplementary Figure 11. a Fragment mass spectra of cysteine containing tryptic strep-MBP-ROXY9 peptides Representative spectra of cysteine-modified (ne: N-Ethylmaleimide, gl: gluathion, tr: sulfonic acid, cy: disulfide) peptides are presented including the used fragmentation method (CID: collision-induced-dissociation, HCD: higher-energy collisional dissociation), the identification score from the search engine MaxQuant and the mass of the isolated precursor ion. As no sequence coverage could be observed in some spectra between C21 and C24, the disulfide bride might be located between those two cysteines.

Supplementary Figure 11

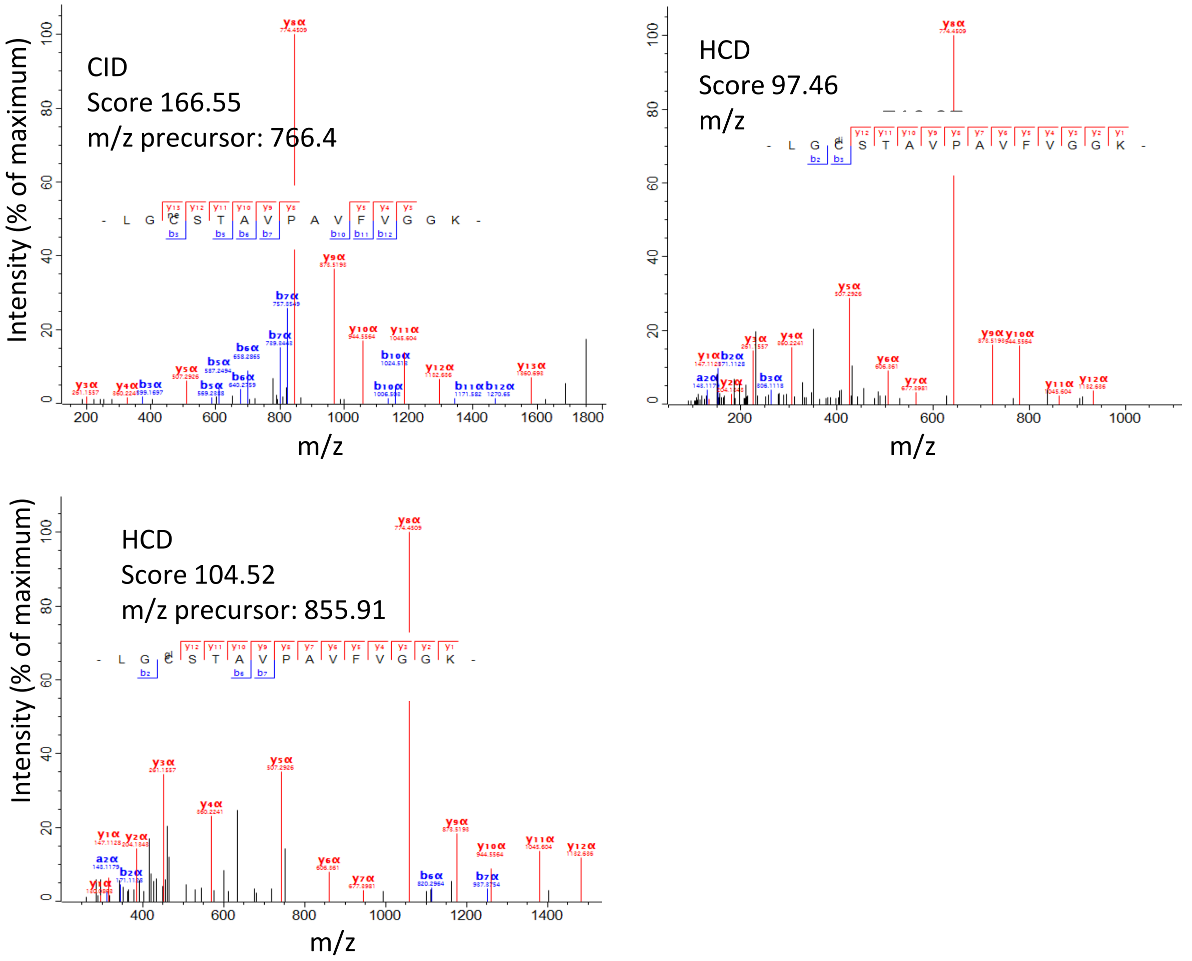

Supplementary Figure 11. b Fragment mass spectra of cysteine containing tryptic strep-MBP-ROXY9 peptides Representative spectra of cysteine-modified (ne: N-Ethylmaleimide, gl: glutathion, di: sulfinic acid) peptides are presented including the used fragmentation method (CID: collision-induced-dissociation, HCD: higher-energy collisional dissociation), the identification score from the search engine MaxQuant and the mass of the isolated precursor ion.

Supplementary Figure 12

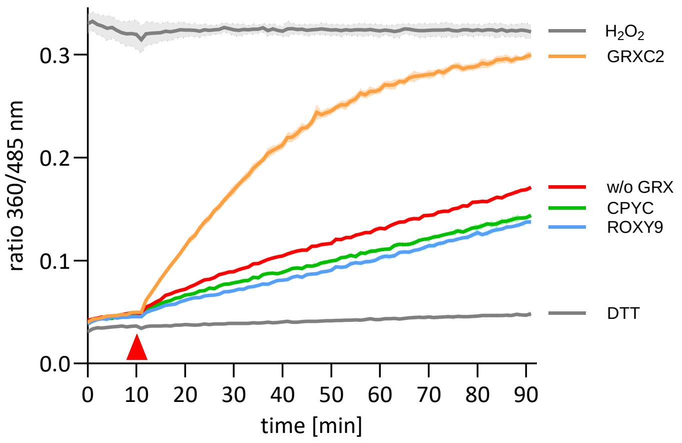

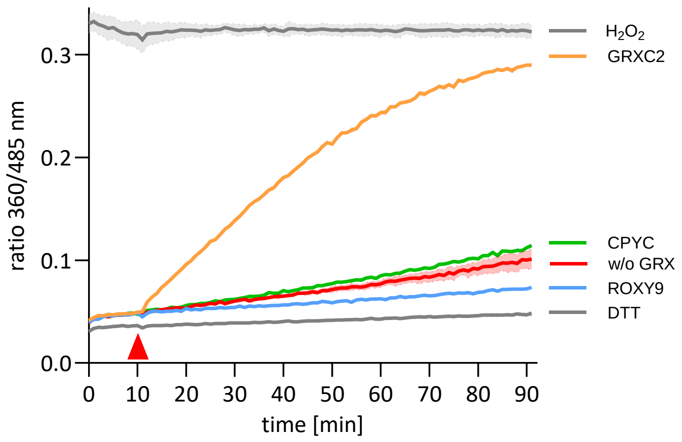

Supplementary Figure 12. roGFP2 assay with strep-MBP-GRXC2, strep-MBP-ROXY9 and strep-MBP-ROXY9-CPYC at -200 mV GSH/GSSG redox potential Final concentrations were 2 µM roGFP2 and 2.5 µM GRX, 50 µM GSSG (upper panel), 11 µM GSSG and 100 µM GSH (lower panel). roGFP2 fluorescence was detected at 528 nm after excitation at 360 and 485 nm. Error bars represent the standard error of the mean resulting from three measurements of one protein preparation (technical replicates). The reaction was performed in 100 mM HEPES, p*H* 7.0, which explains the slower kinetics as compared to Fig. 3 and Supplementary Fig. 7. Values for references (roGFP2 incubated with 10 mM DTT or 10 mM H_2O_2) are the same as in Fig. 3.

Supplementary Figure 13

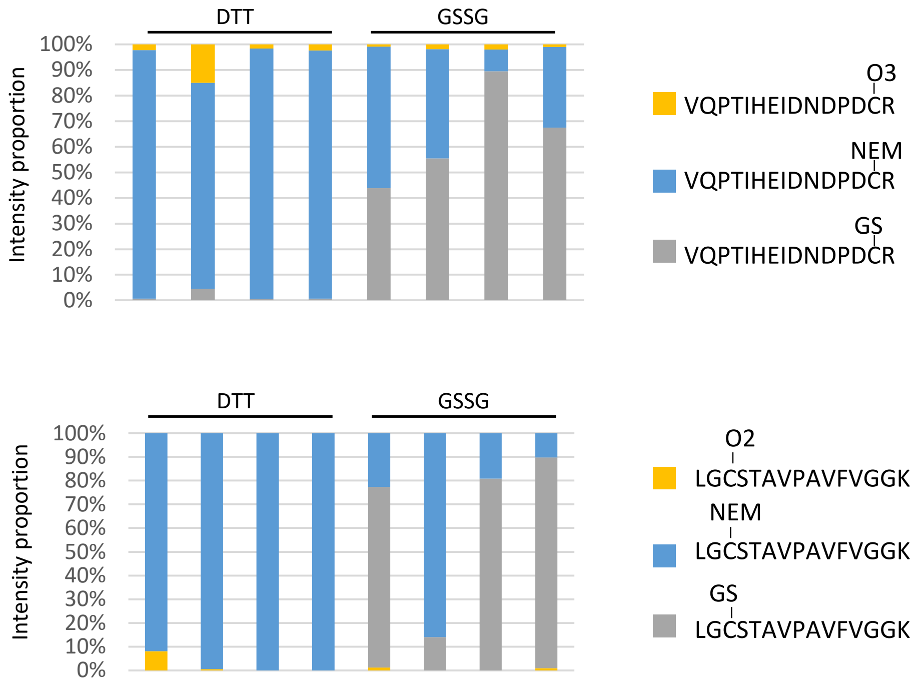

Supplementary Figure 13. Redox modifications of Cys49 and Cys61 of Strep-MBP-ROXY9 Strep-MBP-ROXY9 was pre-reduced with DTT, desalted and mixed with DTT or GSSG, TCA-precipitated, alkylated with NEM and analysed by mass spectrometry. Relative intensity proportions of peptides containing Cys49 (upper panel) or Cys61 (lower panel) with the indicated modifications are shown. The results are from four replicates, with each replicate representing an independent treatment.

Supplementary Figure 14

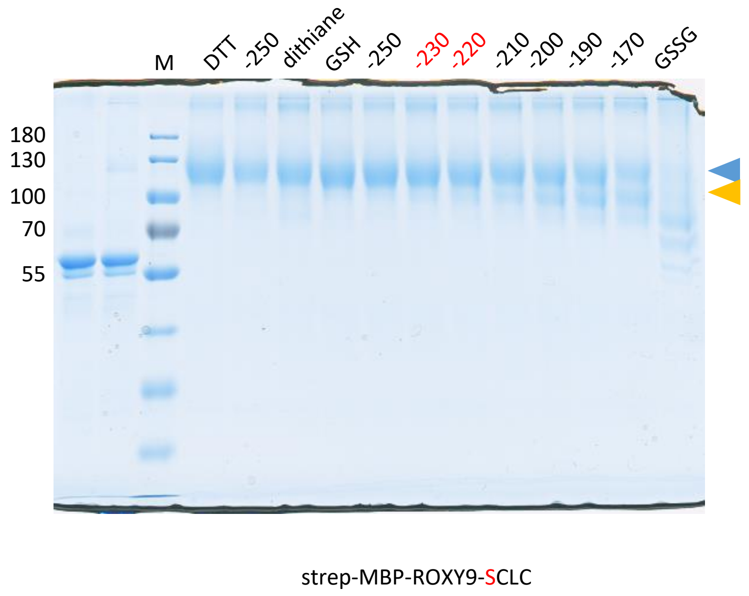

Supplementary Figure 14. Redox titration of active site variant strep-MBP-ROXY9-SCLC Proteins were reduced with 10 mM DTT, desalted and mixed with different DTT/dithiane (10 mM) and GSH/GSSG (10 mM) redox buffers establishing the indicated redox potentials as calculated by the Nernst equation. After TCA precipitation, reduced cysteines were labelled with 5 kDa mmPEG. Samples were separated by non-reducing SDS PAGE. Midpoint redox potentials as observed for the wild-type protein (Fig. 4) are indicated in red. The blue triangle denotes the reduced state, the grey triangle the potentially glutathionylated state. The molecular mass marker was loaded in lane M and sizes in kDa are indicated.

Supplementary Figure 15

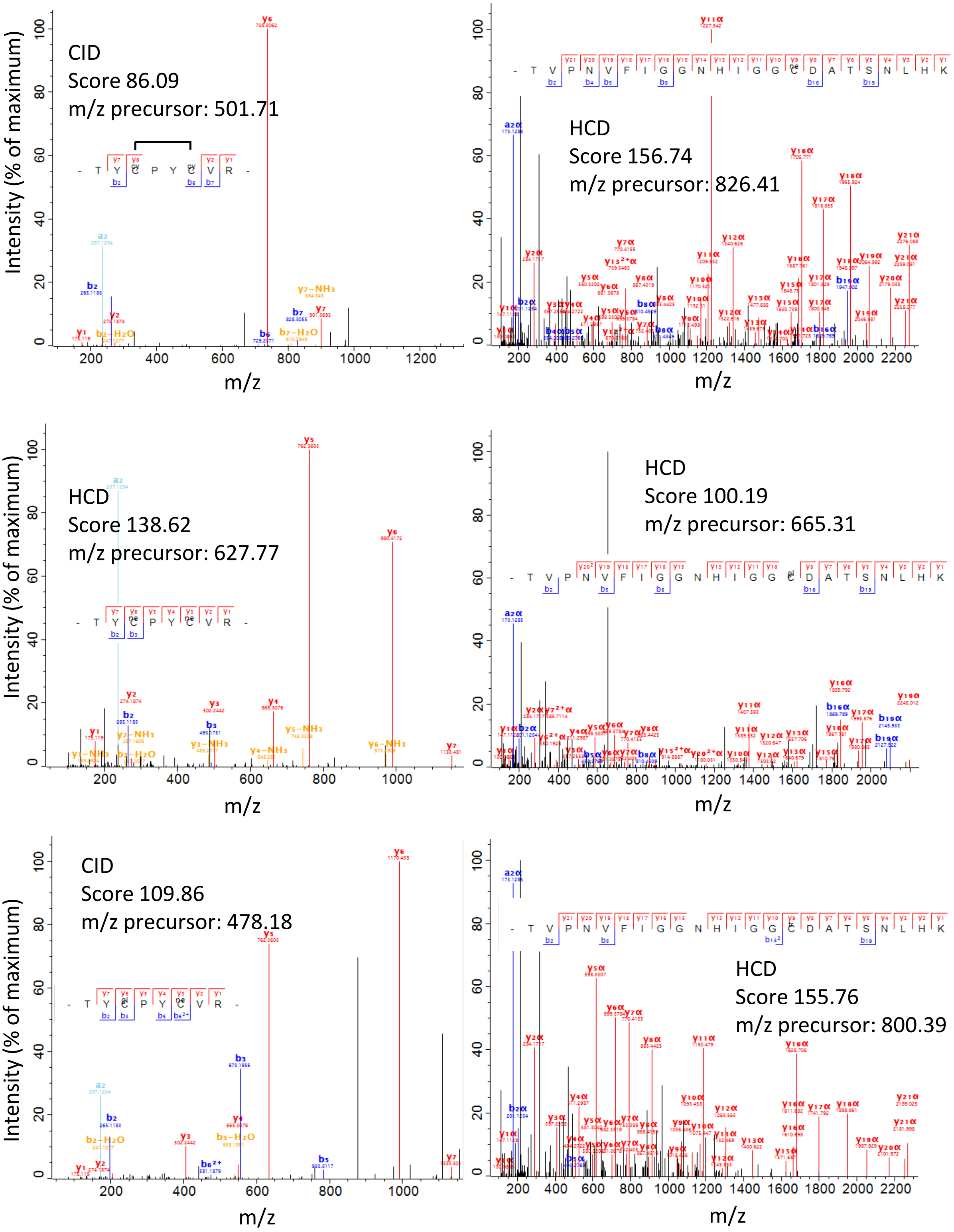

Supplementary Figure 15. Fragment mass spectra of cysteine containing tryptic strep-MBP-GRXC2 peptides Representative spectra of cysteine-modified (ne: N-Ethylmaleimide, gl: glutathion, tr: sulfonic acid, cy: disulfide) peptides are presented including the used fragmentation method (CID: collision-induced-dissociation, HCD: higher-energy collisional dissociation), the identification score from the search engine MaxQuant and the mass of the isolated precursor ion.

Supplementary Figure 16

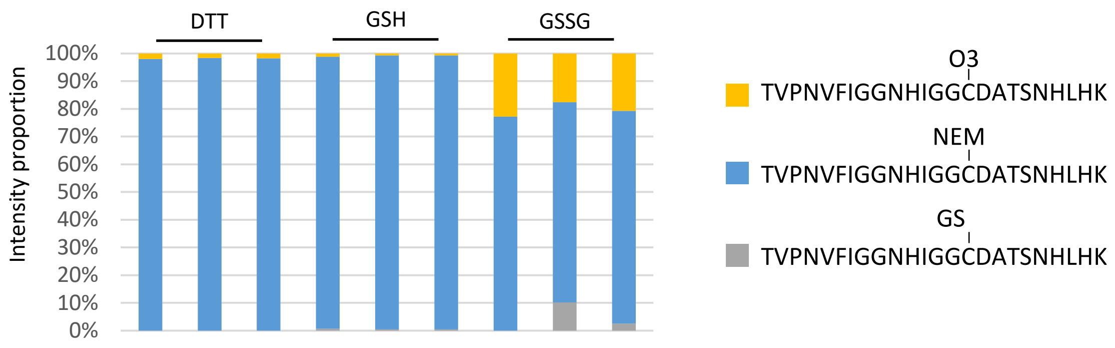

Supplementary Figure 16. Redox modifications of Cys105 of Strep-MBP-GRXC2 Strep-MBP-GRXC2 was pre-reduced with DTT, desalted and mixed with DTT, GSH, or GSSG, TCA-precipitated, alkylated with NEM and analysed by mass spectrometry. Relative intensity proportions of peptides containing Cys105 with the indicated modifications are shown. The results are from three replicates, with each replicate representing an independent treatment.

Supplementary Figure 17

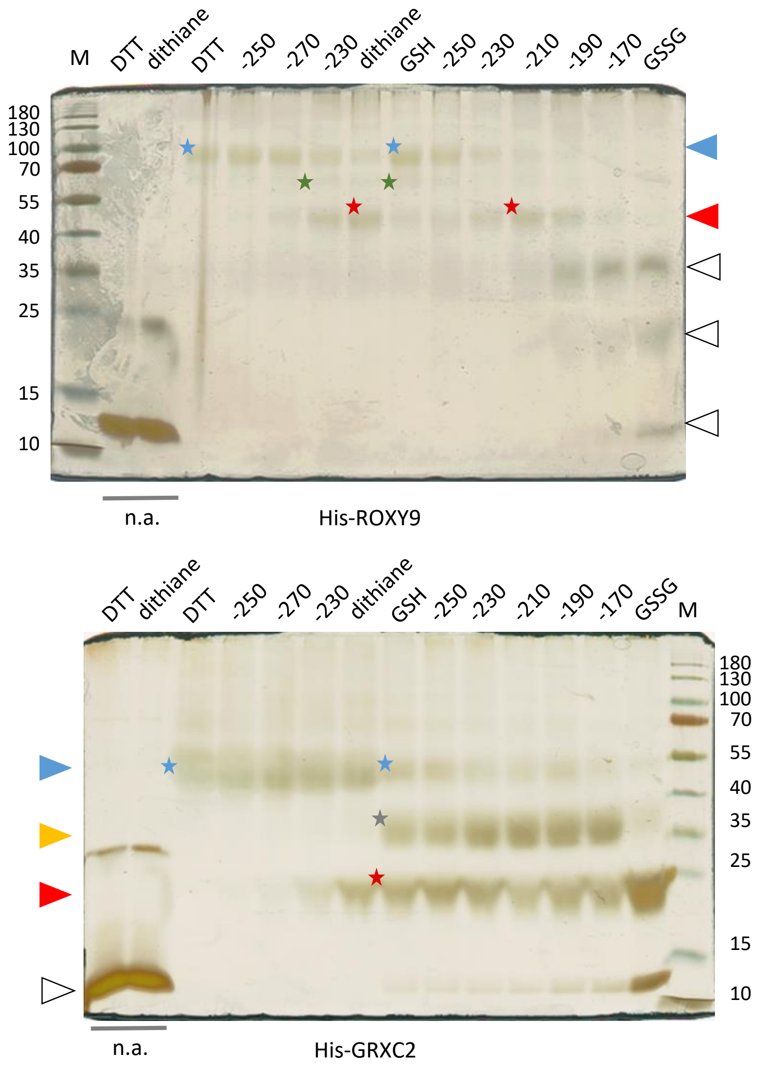

Supplementary Figure 17. Redox titrations of His-ROXY9 and His-GRXC2 His-ROXY9 and His-GRXC2 were pre-reduced with 10 mM DTT, desalted and mixed with DTT/dithiane (10 mM) and GSH/GSSG (10 mM) buffers at the indicated redox potentials as calculated by the Nernst equation, TCA-precipitated, alkylated with mmPEG for analysis on a non-reducing SDS PAGE. Blue triangle/asterisk: fully reduced form, grey triangle/star: glutathionylated at Cys_a, red triangle/asterisk: disulfide bridge between Cys_A and Cys_B, white triangles: glutathionylations at additional cysteine(s), green asterisk: uncharacterized alkylated species. The molecular mass marker was loaded in lanes M and sizes in kDa are indicated. The first lanes (n.a.) contain the respective protein without alkylation. Proteins were stained with silver.

Supplementary Figure 18

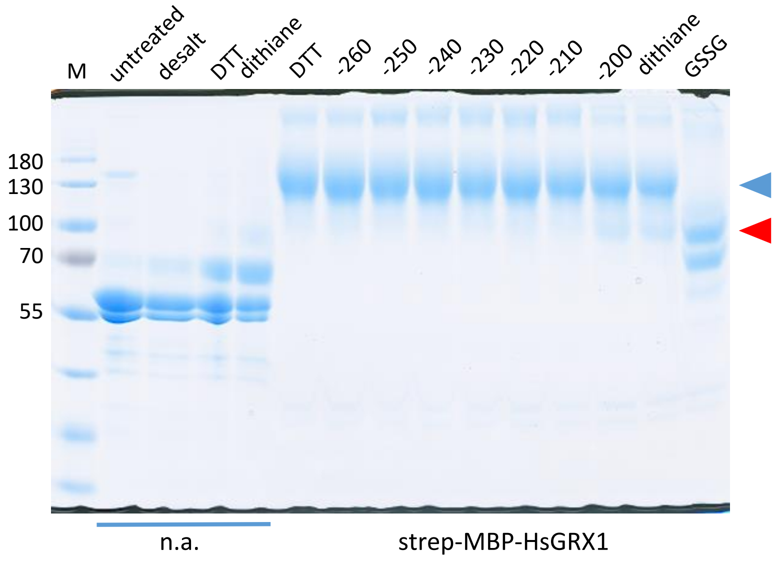

Supplementary Figure 18. Redox titration of strep-MBP-HsGRX1 Strep-MBP-GRX1 was reduced with 10 mM DTT, desalted and mixed with different DTT/dithiane redox buffers (10 mM) establishing the indicated redox potentials or with 10 mM GSSG. After TCA precipitation, reduced cysteines were labelled with 5 kDa mmPEG. Samples were separated by non-reducing SDS PAGE. The first lanes (n.a.) contain the respective protein without alkylation. The "untreated" and "desalted" proteins were loaded without any further manipulation, while the DTT- and dithiane-treated samples were subjected to the same procedure as the other samples but without mmPEG in the alkylation buffer. The blue triangle denotes the reduced protein, the red triangle the disulfide bridge-containing protein. The molecular mass marker was loaded in lane M and sizes in kDa are indicated.

Supplementary Figure 19

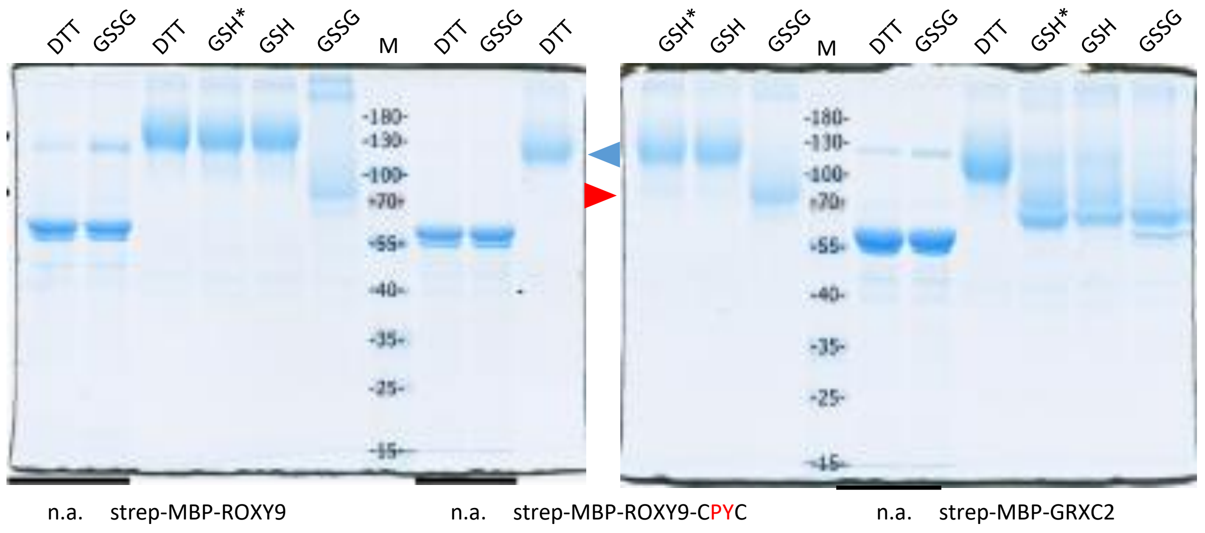

Supplementary Figure 19. Analysis of the redox state of strep-MBP-ROXY9-CPYC Strep-MBP-ROXY-CPYC was reduced with 10 mM DTT, desalted and mixed with 10 mM DTT, 10 mM GSH and 10 mM GSSG as indicated. For the sample marked with GSH*, the desalting column was equilibrated and eluted with 10 mM GSH in order to test for possible oxidation in the absence of any reducing compound. After TCA precipitation, reduced cysteines were labelled with 5 kDa mmPEG. Samples were separated by non-reducing SDS PAGE. The first lanes (n.a.) contain the protein without alkylation. Samples were subjected to the same procedure as the other samples but without mmPEG in the alkylation buffer. The blue triangle denotes the reduced protein, the red triangle the disulfide bridge-containing protein. The molecular mass marker was loaded in lanes M and sizes in kDa are indicated. Samples are from two separate gels.

Supplementary Tables

Supplementary Table 1. Exposed surface areas of cysteines in GRXC2 and ROXY9

AA	volmol	TSA	ESA	rel	#AA-AA	neighbours	#AA-water
**C23**	109,07	136.37	28,19	20.6	9	K20 T21 Y22 P24 Y25 C26 V27 V67 P68	7
**C26**	110,04	137.37	0,60	0.4	13	V17 F18 S19 K20 C23 P24 Y25 V27 R28 V29 K30 V67 P68	2
C80	109,96	136.09	34,08	25.0	9	Y25 R28 V29 P68 G79 D81 A82 T83 S84	7
**C21**	105,53	134.35	48,11	35.8	9	K18 S19 S20 C22 L23 C24 Y25 V65 P66	8
C22	97,94	128.58	84,29	65.6	5	C21 L23 C24 Y25 A26	15
**C24**	107,72	136.02	7,88	0.6	13	I15 F16 T17 K18 C21 C22 L23 Y25 A26 V27 Q28 V65 P66	1
C49	106,82	133.03	31,04	23.3	10	K18 I43 D44 D46 P47 D48 R50 E51 I52 E53	6
C61	113,71	142.52	17,75	12.4	11	L56 L57 L59 G60 S62 T63 P66 A67 L74 G76 S77	2

https://www.dsimb.inserm.fr/dsimb_tools/vldp/index.php

AA: Name and position of the cysteine; volmol: Laguerre/Voronoi Volume of AA; TSA: Total Surface Area of AA; Surface Area Exposed to Solvent; ESA/TSA; number of contacts of AA with other amino acids; AA-WAT: Number of contacts od AA with water molecules. GRXC2: orange, ROXY9: blue. Catalytic and resolving cysteines are indicated in bold, conserved neighbours that contact GSH are underlined. The class III GRX-specific cysteine is highlighted in yellow.

Supplementary Table 2 Sequences of primers used for cloning

**P1/2:** ROXY9 variants for expression in insect cells	fwd. TACTTCCAATCCAATGCAATGGACAAAGTGATGAGAATGTCTTC<br>rev. TTATCCACTTCCAATGTTATTACTAGTAAAGGATGGACTGATAGG
**P3/4:** GRXC2 variants for expression in insect cells	fwd. TACTTCCAATCCAATGCAATGGCGATGCAGAAAGCTAAG<br>rev. TTATCCACTTCCAATGTTATTATTAAGCAGAAGTTGTTGCAGTCTTTC
**P5/6:** HsGRX1 for expression in insect cells	fwd. TACTTCCAATCCAATGCAATGGCTCAAGAGTTTGTGAACTGCAAAATCC<br>rev. TTATCCACTTCCAATGTTATTATTACTGCAGAGCTCCAATCTGCTTTAGC
**P7/8:** roGFP2 for expression in *E. coli*	fwd. GGGGACAAGTTTGTACAAAAAAGCAGGCTCCATGGTGAGCAAGGGCGAG<br>rev. GGGGACCACTTTGTACAAGAAAGCTGGGTTTATTACTTGTACAGCTCGTCCATGCC
**P9/10:** ROXY9 CSLS	fwd. GTTCTCTCTCC TACGCCGTTCAAATCCTGTTC<br>rev. GATTTGAACGGCGTAGGAGAGAGAACATGAGCTCTTCGTGAAGATCACC
**P11/12:** ROXY9 CPYC	fwd. GAAGAGCTCATGTCCATATTGCTACGCCGTTCAAATCCTGTTCCG<br>rev, GGCGTAGCAATATGGACATGAGCTCTTCGTGAAGATCACCACT
**P13/14:** ROXY9 C49S	fwd. CGACAACGACCCGGACTCCCGTGAGATCGAGAAGGC<br>rev. GCCTTCTCGATCTCACGGGAGTCCGGGTCGTTGTCG
**P15/16:** ROXY9 C61S	fwd. CTCTTCTCCGGCTCGGCTCTTCCACGGCGGTTCCAG<br>rev. CTGGAACCGCCGTGGAAGAGCCGAGCCGGAGAAGAG
**P17/18:** GRXC2 CPYS	fwd. GCAAGACTTATTGTCCATATTCCGTGAGAG<br>rev. CTCTCACGGAATATGGACAATAAGTCTTGC
**P19/20:** GRXC2 C80S	fwd. CGGTGGCTCTGATGCAACATCAAACTTG<br>rev. CAAGTTTGATGTTGCATCAGAGCCACCG

blue: extensions for LIC cloning, green: extensions for GATEWAY cloning; red: mutated codon
