# Supplementary material for: Molecular basis for the enzymatic inactivity of class III glutaredoxin ROXY9 on standard glutathionylated substrates
Source: Nat Commun. 2025 Jan 11;16:589. doi: 10.1038/s41467-024-55532-z (PMC11724882; doi:10.1038/s41467-024-55532-z)
Supplement: Supplementary file 2 — Reporting Summary [file 41467_2024_55532_MOESM2_ESM.pdf]

nature portfolio

Corresponding author(s): Gatz

Last updated by author(s): Dec 5, 2024

Reporting Summary

Nature Portfolio wishes to improve the reproducibility of the work that we publish. This form provides structure for consistency and transparency in reporting. For further information on Nature Portfolio policies, see our Editorial Policies and the Editorial Policy Checklist.

Statistics

For all statistical analyses, confirm that the following items are present in the figure legend, table legend, main text, or Methods section.

n/a	Confirmed	
☐	☒	The exact sample size (*n*) for each experimental group/condition, given as a discrete number and unit of measurement
☐	☒	A statement on whether measurements were taken from distinct samples or whether the same sample was measured repeatedly
☒	☐	The statistical test(s) used AND whether they are one- or two-sided<br>*Only common tests should be described solely by name; describe more complex techniques in the Methods section.*
☒	☐	A description of all covariates tested
☒	☐	A description of any assumptions or corrections, such as tests of normality and adjustment for multiple comparisons
☐	☒	A full description of the statistical parameters including central tendency (e.g. means) or other basic estimates (e.g. regression coefficient) AND variation (e.g. standard deviation) or associated estimates of uncertainty (e.g. confidence intervals)
☒	☐	For null hypothesis testing, the test statistic (e.g. *F*, *t*, *r*) with confidence intervals, effect sizes, degrees of freedom and *P* value noted<br>*Give P values as exact values whenever suitable.*
☒	☐	For Bayesian analysis, information on the choice of priors and Markov chain Monte Carlo settings
☒	☐	For hierarchical and complex designs, identification of the appropriate level for tests and full reporting of outcomes
☒	☐	Estimates of effect sizes (e.g. Cohen's *d*, Pearson's *r*), indicating how they were calculated

Our web collection on statistics for biologists contains articles on many of the points above.

Software and code

Policy information about availability of computer code

Data collection	CD Spectroscopy<br>Chirascan Spectrometer Control Panel Application version 4.2.27 (Circular Dichroism)<br>Mass spectrometry<br>Fusion Lumos Tribrid mass spectrometer (Thermo Fisher Scientific) equipped with a nano-electrospray source and FAIMS device<br>roGFP assay<br>Synergy HT; BioTek<br>HED, Cys-SG, GAPDH assays<br>JASCO V-750
Data analysis	CD Spectroscopy<br>Pro-Data Viewer version 4.2.27 (Circular Dichroism)<br>Mass spectrometry<br>MaxQuant version 2.2.0.0<br>Enzymatic assays<br>GraphPad Prism 10

For manuscripts utilizing custom algorithms or software that are central to the research but not yet described in published literature, software must be made available to editors and reviewers. We strongly encourage code deposition in a community repository (e.g. GitHub). See the Nature Portfolio guidelines for submitting code & software for further information.

Data

Policy information about availability of data

All manuscripts must include a data availability statement. This statement should provide the following information, where applicable:

- Accession codes, unique identifiers, or web links for publicly available datasets
- A description of any restrictions on data availability
- For clinical datasets or third party data, please ensure that the statement adheres to our policy

Data that support the findings of this study are available within the paper and its supplementary material. The mass spectrometry proteomics data have been deposited to the ProteomeXchange Consortium via the PRIDE [1] partner repository with the dataset identifier PXD042724 69.Structures are found on https://alphafold.ebi.ac.uk/entry/O82255 (ROXY9) and https://alphafold.ebi.ac.uk/entry/B3H604 (GRXC2). Source data are provided with this paper.

Research involving human participants, their data, or biological material

Policy information about studies with human participants or human data. See also policy information about sex, gender (identity/presentation), and sexual orientation and race, ethnicity and racism.

Reporting on sex and gender	n.a.
Reporting on race, ethnicity, or other socially relevant groupings	n.a.
Population characteristics	n.a.
Recruitment	n.a.
Ethics oversight	n.a.

Note that full information on the approval of the study protocol must also be provided in the manuscript.

Field-specific reporting

Please select the one below that is the best fit for your research. If you are not sure, read the appropriate sections before making your selection.

☒ Life sciences ☐ Behavioural & social sciences ☐ Ecological, evolutionary & environmental sciences

For a reference copy of the document with all sections, see nature.com/documents/nr-reporting-summary-flat.pdf

Life sciences study design

All studies must disclose on these points even when the disclosure is negative.

Sample size	Enzymatic assays (Fig. 3) were performed with three independent protein preparations obtained from three independent insect cell cultures. Critical redox titration experiments were performed with recombinant proteins with a different tag.
Data exclusions	no data were excluded.
Replication	Enzymatic assays (Fig. 3) were performed with three independent protein preparations obtained from three independent insect cell cultures. Critical redox titration experiments were performed with recombinant proteins with a different tag.
Randomization	not applicable, as all data were included in the statistical analysis
Blinding	no blinding, as all data were included in the statistical analysis

Reporting for specific materials, systems and methods

We require information from authors about some types of materials, experimental systems and methods used in many studies. Here, indicate whether each material, system or method listed is relevant to your study. If you are not sure if a list item applies to your research, read the appropriate section before selecting a response.

Materials & experimental systems

n/a	Involved in the study
☒	☐ Antibodies
☐	☒ Eukaryotic cell lines
☒	☐ Palaeontology and archaeology
☒	☐ Animals and other organisms
☒	☐ Clinical data
☒	☐ Dual use research of concern
☒	☐ Plants

Methods

n/a	Involved in the study
☒	☐ ChIP-seq
☒	☐ Flow cytometry
☒	☐ MRI-based neuroimaging

Eukaryotic cell lines

Policy information about cell lines and Sex and Gender in Research

Cell line source(s)	Triplusia ni Hi5, Spodoptera frugiperda Sf-9
Authentication	n.a.
Mycoplasma contamination	n.a.
Commonly misidentified lines (See ICLAC register)	n.a.

Plants

Seed stocks	n.a.
Novel plant genotypes	n.a.
Authentication	n.a.
